# 548 Volatile Sedation in the Burn Intensive Care Unit: A Quality Improvement Initiative

**DOI:** 10.1093/jbcr/irad045.145

**Published:** 2023-05-15

**Authors:** Julie Nardi, Lauren Wong, Alan Rogers

**Affiliations:** Ross Tilley Burn Centre, Sunnybrook Health Sciences Centre, Toronto, Ontario; University of Ottawa, Ottawa, Ontario; Ross Tilley Burn Centre, University of Toronto, Toronto, Ontario; Ross Tilley Burn Centre, Sunnybrook Health Sciences Centre, Toronto, Ontario; University of Ottawa, Ottawa, Ontario; Ross Tilley Burn Centre, University of Toronto, Toronto, Ontario; Ross Tilley Burn Centre, Sunnybrook Health Sciences Centre, Toronto, Ontario; University of Ottawa, Ottawa, Ontario; Ross Tilley Burn Centre, University of Toronto, Toronto, Ontario

## Abstract

**Introduction:**

Achieving optimal sedation and analgesia in patients with major burn injuries can be challenging. Reliance on benzodiazepines results in longer durations of mechanical ventilation, delirium and withdrawal. Recent evidence and local experience in our critical care unit has supported the application of volatile agents such as isoflurane for sedation. A local audit determined that 70% of mechanically ventilated patients in our burn center are managed on 3 or more continuous sedative infusions. The aim of this initiative was to reduce the number of continuous sedative infusions to maintain target sedation range (SAS 2-4) by 25% in the 48 hour period following initiation of isoflurane.

**Methods:**

A multifaceted education approach was applied to engage all bedside staff, including burn surgeons, respiratory therapists and nurses. Training occurred though self-directed modules, in-person learning and just-in-time training upon new patient initiation. Data from the 18 month period since implementation were collected retrospectively via manual chart review.

**Results:**

Since implementation in March 2021, 18 patients were sedated using isoflurane in our burn intensive care unit (ICU). Within 48 hours of isoflurane initiation, 50% (9/18) of patients had a decrease in the number of sedative infusions and 56% (10/18) obtained an optimal level of sedation using isoflurane and hydromorphone alone.

**Conclusions:**

Volatile agents provide a safe and reliable method of sedation in mechanically ventilated patients with major burn injuries. Despite limitations related to specialized equipment and training, the introduction of this strategy has been well received, with positive feedback relating to both ease of implementation and titration.

**Applicability of Research to Practice:**

Patients with major burn injuries require multiple painful surgeries and dressing changes. Volatile sedation has been shown to reduce the number of sedative infusions, specifically, our reliance on benzodiazepines.

